# Successful management of delayed presentation of massive paracetamol overdose in a resource‐limited setting: A case report from Nepal

**DOI:** 10.1002/ccr3.6733

**Published:** 2022-12-12

**Authors:** Pratik Lamichhane, Kailash Mani Pokhrel, Bijay Bhandari, Anushka Agrawal, Bhumika Ghimire, Olita Shilpakar

**Affiliations:** ^1^ Maharajgunj Medical Campus Institute of Medicine Kathmandu Nepal; ^2^ Drug and Patient Safety Unit Lumbini Provincial Hospital Rupandehi Nepal; ^3^ Department of Emergency Medicine, TUTH Kathmandu Nepal

**Keywords:** acetaminophen, acetylcysteine, case report, Nepal, poisoning

## Abstract

We present a case of self‐poisoning with a massive dose of paracetamol by a young Nepalese female patient who presented late to our emergency department. This report highlights the successful management of the patient with the extended use of N‐acetylcysteine over 4 days and continuous supportive therapy as required. The case is an example of the management of delayed presentation of a massive paracetamol poisoning in a resource‐limited setting, where intensive care units and hemodialysis facilities are not easily available. However, when available, massive poisoning should always be managed in continuous monitoring units under the expertise of a toxicologist.

## 1 INTRODUCTION

Paracetamol or acetaminophen is an over‐the‐counter antipyretic and analgesic drug notorious for intentional and accidental self‐poisoning. Paracetamol poisoning accounts for the majority of overdoses in Europe, North America, and Australia.^1^, ^2^Paracetamol was also the single most used drug for acute poisoning in India.^3^ Paracetamol poisoning can be fatal owing to its hepatotoxic and nephrotoxic nature.^4^, ^5^

Acute ingestion of at least 7.5–10 gms in adults or 150–200 mg/kg in children above 6 years is considered a toxic dose of paracetamol, which is likely to produce hepatotoxicity.^6^A paracetamol overdose is considered massive when the intake of paracetamol is ≥30 grams.^7^ Acute ingestion is defined as any intentional or deliberate paracetamol overdose, including staggered or multiple paracetamol ingestions over more than 2 h. ^2^ N‐acetylcysteine (NAC) is the antidote of choice in paracetamol poisoning. Early use of activated charcoal, an increased dose of NAC, and early hemodialysis are suggested for a massive overdose of paracetamol.^8^, ^9^

Herein, we present a 24‐year‐old patient who presented 40 h after the consumption of 50 grams of paracetamol. Despite delayed presentation, the patient was managed with the prolonged use of NAC and continuous supportive therapy without extracorporeal treatment, which sets this case apart from others.

## 2 CASE PRESENTATION

A 24‐year‐old female patient presented to the Emergency Department (ED) of Tribhuvan University Teaching Hospital (TUTH) at 6:45 a.m. on December 13, 2021 with an alleged history of ingestion of paracetamol tablets about 40 h ago. Our risk assessment revealed that the patient consumed a total of 100 tablets, each containing 500 mg (total = 50 grams), over 4 h. Different formulations, such as immediate‐release and delayed‐release, in unmeasured quantities, were supposedly consumed by the patient. After 6 h of acute single ingestion, she developed nausea and vomiting, along with abdominal pain. The patient was immediately rushed to a nearby local hospital where routine laboratory investigations were performed and symptomatic management was started. An intravenous (IV) line was opened to provide fluids, pantoprazole 40 mg, ondansetron 2 mg, and ceftriaxone 1gm. She received a referral to TUTH for further management due to the unavailability of antidote N‐acetyl cysteine (NAC) and intensive care unit (ICU) for monitoring. The reason behind her intentional overdose was not clear but she pointed toward the whole act being performed out of curiosity. She had planned the ingestion for a month before the event. During this time, she bought 100 tablets from nearby pharmaceutical dispensaries.

The patient had no history of co‐morbidities requiring her to consume paracetamol. Similarly, she had no known psychiatric problems, and this was her first act of self‐harm. Her last menstruation was 2 days before the event. Her vital status was as follows: pulse rate: 74 beats/minute, blood pressure: 110/60 mmHg, temperature: 36.7°C, respiratory rate: 20/minute, and O_2_ saturation: 99%. The patient was awake, alert, and well oriented to time, place, and person. The patient's general condition was fair, with mild pallor but no icterus, edema, or dehydration. Her per abdominal examination revealed generalized abdominal tenderness, predominant in the left iliac and left hypochondrium regions. However, other systemic examinations were unremarkable. The results of laboratory tests at admission to our ED (about 40 h post‐ingestion) are shown in Table 1.

**TABLE 1:** Laboratory investigations of the patient at admission

Laboratory tests	Patient's values	Reference values
Hemoglobin(gm/dl)	9.6	12.5–15.0
Packed Cell Volume (%)	30	37.5–45
RBC (million/mm^3^)	3.60	3.5–4.5
WBC(/mm^3^)	4100	4000–11,000
Neutrophils (%)	76	45–75
Lymphocytes (%)	20	25–45
Monocytes (%)	4	2–10
Eosinophils (%)	0	1–6
Basophils (%)	0	0–1
Platelets (/mm^3^)	124,000	150,000–400,000
Random blood sugar (mmol/L)	4.8	3.8–7.8
Urea (mmol/L)	5.0	1.6–7.0
Creatinine (uMol/L)	60	40–110
Total bilirubin (uMol/L)	33.0	3–21
Direct bilirubin (uMol/L)	10.0	4
SGPT/ALT (U/L)	125	42
SGOT/AST (U/L)	130	37
Alkaline phosphatase (U/L)	68.0	30–90
Gamma GT (U/L)	10.0	11–50
Total protein (gm/L)	67.0	60–80
Albumin (gm/L)	39.0	37–47
Amylase (U/L)	55.0	80
PT (seconds)	20	10–12
PT INR	1.66	0.8–1.1
pH	7.42	7.35–7.45
HCO3 (mmol/L)	21.2	22–26
PCO2 (mmHg)	32.5	35–45
Serum paracetamol (ugm/ml)	14.2	10.0–30.0

Her initial investigations revealed anemia, thrombocytopenia, deranged liver function tests (LFTs), a raised amylase level, and slightly raised PT INR. Her serum paracetamol concentration was at 14.2 ugm/ml which was within the therapeutic range. Her urinalysis revealed hematuria (22–24/high power field) with trace albumin. Her electrocardiogram and ultrasonography of her abdomen and pelvis showed normal findings.

At our ED, she received IV pantoprazole 40 mg and IV ondansetron 4 mg stat. In addition to this, she (weight = 40 kgs) also received IV NAC 6 gm (dose = 150 mg/kg) in 500 ml dextrose normal saline (DNS) over 30 min. Next, 2 gm (dose = 50 mg/kg)IV NAC was infused over 4 h. Finally, 4 gm (dose = 100 mg/kg) NAC was started for 16 h as a final dose of 21 h IV protocol.^10^ She also received an IV infusion of 10 ml L‐Ornithine‐L‐Aspartate(LOLA) over 8 h. Furthermore, an injection of vitamin K 10 mg was also administered.

On the second day of hospitalization, she was shifted to the observation unit of the ED. The vitals were within normal range. However, she had icterus and mild generalized tenderness of the abdomen. Her liver enzymes, ALT and AST, were about eight times higher than the values on the first day of admission. Appropriate symptomatic management was provided along with an infusion of 10 ml of LOLA on the second day. Her vitals remained stable throughout her stay in the hospital. The LFTs were assessed every day of her stay in the hospital. The results of LFTs are presented in Table 2.

**TABLE 2:** Serial liver function tests values of the patient

Investigations	Day 1	Day 2	Day 3	Day 4	Day 5
Total bilirubin (uMol/L)	33	18	19	12	10
Direct bilirubin (uMol/L)	10	3	6	3	2
SGPT/ALT (U/L)	125	910	1150	591	474
SGOT/AST (U/L)	130	980	565	220	131
Alkaline phosphatase (U/L)	68	75	65	76	80
Total protein (gm/L)	67	65	58	56	56
Albumin (gm/L)	39	36	35	35	34
PT (sec)	20	19.2	17	15	12
PT INR	1.66	1.6	1.41	1.25	1.0

**FIGURE 1:**
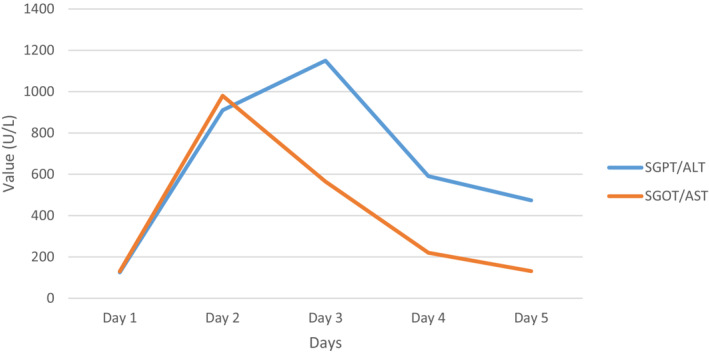
Monitoring of ALT and AST since the admission of the patient

On the third day, her ALT level was the highest of all at 1150 U/L. On this background of high and increasing ALT levels, NAC infusion was resumed since it was stopped after completion of the 21 h treatment protocol. Therefore, an IV infusion of 4 gm of NAC was given to the patient over the course of 12 h. The same dose of NAC was continued for the next 3 days of her stay. The IV infusion of LOLA was also continued for the next 3 days. With persistent treatment, her clinical status and LFTs improved, and thus, NAC treatment was stopped. The pattern of liver enzymes throughout the hospital stay is shown in Figure 1. On the sixth day, a psychiatric consultation was done, which gave an impression of a suicidal attempt and borderline personality traits. The patient was discharged well on the seventh day of the hospital stay. The patient was advised to follow‐up at the outpatient clinic of the psychiatry department for further management. A written informed consent was obtained from the patient for the publication of this report.

## 3 DISCUSSION

Paracetamol is an effective antipyretic and analgesic that has been used comprehensively and globally for many years. The exact mechanism of action of paracetamol is not yet clearly understood. However, complex mechanisms involving cyclooxygenases, endocannabinoid system, serotonergic pathways, and nitric oxide synthesis pathways have been discussed in the literature.^11^

Paracetamol at the therapeutic dose (up to 4 gm/day for adults and 50–75 mg/kg/day in children) has a safety profile, but an overdose can induce life‐threatening adverse reactions like acute hepatic failure.^1^, ^12^ Under the recommended therapeutic doses, 90% of paracetamol is conjugated in the liver into sulfate and glucuronide metabolites, which are non‐toxic and readily excreted by the kidneys. A small fraction (5%–10%) of acetaminophen is metabolized by CYP450 isoforms to a toxic and highly reactive metabolite: N‐acetyl‐p‐benzoquinoneimine (NAPQI). This reactive compound is detoxified in hepatocytes by conjugation with glutathione. When a very large dose of paracetamol (≥150 mg/kg or >10 g in an adult) is taken, the glucuronidation capacity of the liver is saturated. Therefore, more of the NAPQI binds covalently with protein in the hepatocytes and tubular cells of the kidneys, causing hepatic necrosis and acute tubular necrosis, respectively.^12^

Paracetamol poisoning leads to acute renal failure with oliguria and deranged renal function tests.^5^ Moreover, massive overdoses have led to central nervous system depression and metabolic acidosis.^13^ Paracetamol is one of the most commonly reported products causing drug‐induced liver injury.^12^, ^14^, ^15^ Easy availability, the inclusion of paracetamol in numerous drug combinations, lack of knowledge regarding toxicity, and misunderstanding regarding dosing patterns are the reasons behind frequent encounters of paracetamol poisoning in health settings.^16^

IV NAC is the widely accepted antidote for paracetamol poisoning and is very effective when used early in the course of poisoning.^17^ Administration of NAC, even in delayed presentations with evidence of hepatic failure, decreased mortality and improved hepatic and cerebral function in the patients.^18^, ^19^Several international guidelines are available regarding treatment with NAC. These guidelines differ in threshold paracetamol concentrations for the initiation of treatment and the duration of treatment. Our patient was treated initially in the lines of a 21 h IV protocol with a total dose of 12 grams. The 21 h, three‐bag NAC treatment regimen is considered standard therapy and has been widely used.^10^ A new guideline recommends a simplified two‐bag NAC regimen (200 mg/kg over 4 h, then 100 mg/kg over 16 h) owing to similar efficacy and fewer adverse reactions compared to standard therapy. The guideline also recommends treating massive paracetamol overdoses with an increased dose and higher infusion of rates of NAC.^2^

This case management was challenging to us in many aspects. Since the patient presented after 40 h of ingestion of a massive dose, the Rumack–Mathew nomogram could not be used to guide the initiation of treatment.^20^ The serum paracetamol level peaks at 4 h after oral intake and may no longer be detectable in a delayed presentation because of drug metabolism and excretion.^12^ Our patients' serum paracetamol level was within the therapeutic range at admission. Since we could not accurately quantify the amount of the drug consumed, the low serum drug level could have been due to consumption in a lower quantity than given in history. Furthermore, the patient had consumed different preparations of paracetamol, which led to a dilemma in opting for decontamination procedures. However, the treating team decided that such procedures would offer no benefit considering her delayed presentation. Similarly, choosing an appropriate treatment regimen after the completion of one 21 h infusion was also challenging in this case. It is recommended to continue infusion at rates upto 200 mg/kg in cases of massive overdoses.^2^ Since the serum paracetamol concentration was within the therapeutic range, NAC infusion was continued for three more days at a lower rate of 100 mg/kg in this patient.

Massive paracetamol overdoses have been reported occasionally in the literature, with variable outcomes. Two case reports have described patients who were treated successfully with intravenous or oral preparations of NAC for three continuous days without significant adverse reactions.^21^, ^22^ On the contrary, some studies have described the development of hepatotoxicity and liver failure despite early administration of the antidote.^23^, ^24^Similarly, mortality with massive poisoning has also been described in the literature. One of such reports described a patient who succumbed to cardiac arrest despite aggressive treatment.^25^

Our patient has received multiple infusions of LOLA which gets converted into glutathione and replaces the depleted source in the liver created by paracetamol overdose. LOLA has been shown to correct the glutathione loss with liver failure resulting from toxic liver injury.^26^, ^27^Our patient did not develop acute liver failure despite a massive overdose and a long delay between ingestion and antidote administration. The pharmacogenetics of paracetamol metabolism of the patient might have played a role in the favorable prognosis. A very small portion of the Asian population developed hepatotoxicity and ALF despite high doses and delayed presentations, according to a study by Marzilawati et al.^28^ Vigilant supportive treatment along with repeated NAC treatment must have aided in the overall good prognosis.

## 4 CONCLUSIONS

This case highlights that recovery is possible in massive paracetamol poisoning with extended NAC treatment for days in resource‐limited settings where ICU or hemodialysis facilities are unavailable. Therefore, NAC should be started as soon as possible under the suspicion of significant ingestion despite delayed presentation. However, when available, patients should always be managed in the continuous monitoring units by a team with expertise in managing drug overdoses. Furthermore, this report has shed light on the ease of accessibility to large amounts of over‐the‐counter medications for the general public in Nepal. Public health measures that restrict the availability of paracetamol, such as reducing non‐prescription pack sizes, are needed to stanch such cases of massive paracetamol overdoses in Nepal.

## AUTHOR CONTRIBUTIONS

**Pratik Lamichhane:** Conceptualization; data curation; methodology; writing – original draft; writing – review and editing. **Kailash Mani Pokhrel:** Data curation; writing – original draft. **Bijay Bhandari:** Writing – original draft. **Anushka Agrawal:** Writing – original draft. **Bhumika Ghimire:** Writing – original draft. **Olita Shilpakar:** Writing – review and editing.

## CONFLICT OF INTEREST

None declared.

## CONSENT

Written informed consent was obtained from the patient to publish this report in a accordance with the journal's patient consent policy.

## Data Availability

The data that support the findings of this study are available from the corresponding author upon reasonable request.

## References

[1] Lancaster EM , Hiatt JR , Zarrinpar A . Acetaminophen hepatotoxicity: an updated review. Arch Toxicol. 2015;89(2):193‐199. doi:10.1007/s00204-014-1432-2 25537186

[2] Chiew AL , Reith D , Pomerleau A , et al. Updated guidelines for the management of paracetamol poisoning in Australia and New Zealand. Med J Australia. 2020;212(4):175‐183. doi:10.5694/mja2.50428 31786822

[3] Nair SJ , Sujatha C , Chettiar KPS , Sasikala K . Toxico‐epidemiology of acute poisoning; an exploratory study from a tertiary care hospital in South India along with global comparisons and solutions. J Forensic Leg Med. 2021;83:102247. doi:10.1016/j.jflm.2021.102247 34454338

[4] Tittarelli R , Pellegrini M , Scarpellini MG , et al. Hepatotoxicity of paracetamol and related fatalities. Eur Rev Med Pharmacol Sci. 2017;21(1 Suppl):95‐101. 28379590

[5] O'Riordan A , Brummell Z , Sizer E , et al. Acute kidney injury in patients admitted to a liver intensive therapy unit with paracetamol‐induced hepatotoxicity. Nephrol Dial Transplant. 2011;26(11):3501‐3508. doi:10.1093/ndt/gfr050 21652548

[6] Hodgman MJ , Garrard AR . A review of acetaminophen poisoning. Crit Care Clin. 2012;28(4):499‐516. doi:10.1016/j.ccc.2012.07.006 22998987

[7] Marks DJB , Dargan PI , Archer JRH , et al. Outcomes from massive paracetamol overdose: a retrospective observational study. Br J Clin Pharmacol. 2017;83(6):1263‐1272. doi:10.1111/bcp.13214 28002875 PMC5427245

[8] Chiew AL , Isbister GK , Kirby KA , Page CB , Chan BSH , Buckley NA . Massive paracetamol overdose: an observational study of the effect of activated charcoal and increased acetylcysteine dose (ATOM‐2). Clin Toxicol. 2017;55(10):1055‐1065. doi:10.1080/15563650.2017.1334915 28644687

[9] Gosselin S , Juurlink DN , Kielstein JT , et al. Extracorporeal treatment for acetaminophen poisoning: recommendations from the EXTRIP workgroup. Clin Toxicol. 2014;52(8):856‐867. doi:10.3109/15563650.2014.946994 25133498

[10] Prescott LF , Illingworth RN , Critchley JA , Stewart MJ , Adam RD , Proudfoot AT . Intravenous N‐acetylcystine: the treatment of choice for paracetamol poisoning. Br Med J. 1979;2(6198):1097‐1100. doi:10.1136/bmj.2.6198.1097 519312 PMC1597048

[11] Przybyła GW , Szychowski KA , Gmiński J . Paracetamol – an old drug with new mechanisms of action. Clin Exp Pharmacol Physiol. 2021;48(1):3‐19. doi:10.1111/1440-1681.13392 32767405

[12] Bunchorntavakul CMD , Reddy KRMD . Acetaminophen‐related Hepatotoxicity. Clin Liver Dis. 2013;17(4):587‐607. doi:10.1016/j.cld.2013.07.005 24099020

[13] Flanagan RJ , Mant TGK . Coma and metabolic acidosis early in severe acute paracetamol poisoning. Hum Toxicol. 1986;5(3):179‐182. doi:10.1177/096032718600500305 3710495

[14] Yoon E , Babar A , Choudhary M , Kutner M , Pyrsopoulos N . Acetaminophen‐induced Hepatotoxicity: a comprehensive update. J Clin Transl Hepatol. 2016;4(2):131‐142. doi:10.14218/JCTH.2015.00052 27350943 PMC4913076

[15] Clark R , Fisher JE , Sketris IS , Johnston GM . Population prevalence of high dose paracetamol in dispensed paracetamol/opioid prescription combinations: an observational study. BMC Clin Pharmacol. 2012;12:11‐11. doi:10.1186/1472-6904-12-11 22709372 PMC3416683

[16] Herndon CM , Dankenbring DM . Patient perception and knowledge of acetaminophen in a large family medicine service. J Pain Palliat Care Pharmacother. 2014;28(2):109‐116. doi:10.3109/15360288.2014.908993 24813653

[17] Chiew AL , Gluud C , Brok J , Buckley NA . Interventions for paracetamol (acetaminophen) overdose. Cochrane Database Syst Rev. 2018;2(2):Cd003328. doi:10.1002/14651858.CD003328.pub3 29473717 PMC6491303

[18] Keays R , Harrison PM , Wendon JA , et al. Intravenous acetylcysteine in paracetamol induced fulminant hepatic failure: a prospective controlled trial. Br Med J. 1991;303(6809):1026‐1029. doi:10.1136/bmj.303.6809.1026 1954453 PMC1671790

[19] Harrison PM , Keays R , Bray GP , Alexander GJM , Williams R . Improved outcome of paracetamol‐induced fulminant hepatic failure by late administration of acetylcysteine. The Lancet. 1990;335(8705):1572‐1573. doi:10.1016/0140-6736(90)91388-Q 1972496

[20] Rumack BH , Matthew H . Acetaminophen poisoning and toxicity. Pediatrics. 1975;55(6):871‐876. 1134886

[21] Sule AA , Tai DY , Tze CC , Deepa B , Leow MK . Potentially fatal paracetamol overdose and successful treatment with 3 days of intravenous N‐acetylcysteine regime‐‐a case report. Ann Acad Med Singapore. 2006;35(2):108‐111. 16565765

[22] Benlamkaddem S , Iken I , Houari N , et al. Paracetamol self‐poisoning: when oral N‐acetylcysteine saves life? a case report. Pan Afr Med J. 2018;29:83‐83. doi:10.11604/pamj.2018.29.83.10595 29875964 PMC5987070

[23] Schwartz EA , Hayes BD , Sarmiento KF . Development of hepatic failure despite use of intravenous acetylcysteine after a massive ingestion of acetaminophen and diphenhydramine. Ann Emerg Med. 2009;54(3):421‐423. doi:10.1016/j.annemergmed.2008.10.001 18986731

[24] Doyon S , Klein‐Schwartz W . Hepatotoxicity despite early Administration of Intravenous N‐acetylcysteine for acute acetaminophen overdose. Acad Emerg Med. 2009;16(1):34‐39. doi:10.1111/j.1553-2712.2008.00296.x 19007345

[25] Stead TS , Jeong JY , Ganti L , Rubero J . Massive acetaminophen overdose. Cureus. 2020;12(7):e9262‐e9262. doi:10.7759/cureus.9262 32821608 PMC7431296

[26] Butterworth RF , Canbay A . Hepatoprotection by L‐ornithine L‐aspartate in non‐alcoholic fatty liver disease. Dig Dis. 2019;37(1):63‐68. doi:10.1159/000491429 30016770 PMC6390461

[27] Najmi AK , Pillai KK , Pal SN , Akhtar M , Aqil M , Sharma M . Effect of l‐ornithine l‐aspartate against thioacetamide‐induced hepatic damage in rats. Indian J Pharmacol. 2010;42(6):384‐387. doi:10.4103/0253-7613.71926 21189911 PMC2991698

[28] Marzilawati A‐R , Ngau Y‐Y , Mahadeva S . Low rates of hepatotoxicity among Asian patients with paracetamol overdose: a review of 1024 cases. BMC Pharmacol Toxicol. 2012;13(1):8. doi:10.1186/2050-6511-13-8 23021009 PMC3517419

